# Risk Assessment for Parents Who Suspect Their Child Has Autism Spectrum Disorder: Machine Learning Approach

**DOI:** 10.2196/jmir.9496

**Published:** 2018-04-24

**Authors:** Ayelet Ben-Sasson, Diana L Robins, Elad Yom-Tov

**Affiliations:** ^1^ Department of Occupational Therapy, Faculty of Social Welfare and Health Sciences University of Haifa Haifa Israel; ^2^ AJ Drexel Autism Institute Drexel University Philadelphia, PA United States; ^3^ Microsoft Research Herzelia Israel

**Keywords:** autistic disorder, early diagnosis, screening, parents, child, expression of concern, technology, machine learning

## Abstract

**Background:**

Parents are likely to seek Web-based communities to verify their suspicions of autism spectrum disorder markers in their child. Automated tools support human decisions in many domains and could therefore potentially support concerned parents.

**Objective:**

The objective of this study was to test the feasibility of assessing autism spectrum disorder risk in parental concerns from Web-based sources, using automated text analysis tools and minimal standard questioning.

**Methods:**

Participants were 115 parents with concerns regarding their child’s social-communication development. Children were 16- to 30-months old, and 57.4% (66/115) had a family history of autism spectrum disorder. Parents reported their concerns online, and completed an autism spectrum disorder-specific screener, the Modified Checklist for Autism in Toddlers-Revised, with Follow-up (M-CHAT-R/F), and a broad developmental screener, the Ages and Stages Questionnaire (ASQ). An algorithm predicted autism spectrum disorder risk using a combination of the parent's text and a single screening question, selected by the algorithm to enhance prediction accuracy.

**Results:**

Screening measures identified 58% (67/115) to 88% (101/115) of children at risk for autism spectrum disorder. Children with a family history of autism spectrum disorder were 3 times more likely to show autism spectrum disorder risk on screening measures. The prediction of a child’s risk on the ASQ or M-CHAT-R was significantly more accurate when predicted from text combined with an M-CHAT-R question selected (automatically) than from the text alone. The frequently automatically selected M-CHAT-R questions that predicted risk were: following a point, make-believe play, and concern about deafness.

**Conclusions:**

The internet can be harnessed to prescreen for autism spectrum disorder using parental concerns by administering a few standardized screening questions to augment this process.

## Introduction

### Challenges of Early Autism Spectrum Disorder Screening

Many parents of children with autism spectrum disorder (ASD) report their concerns when their child is 12- to 19-month old [1,2], yet the average age of ASD diagnosis is above 3 years [1]. Evidence shows that 5 to 6 months, on average, pass from when the parent of a child with ASD becomes concerned until they approach a professional. Moreover, over 32 months pass until a diagnosis is made [3]. This delay wastes a critical time period, during which targeted intensive intervention is most efficient [4]; the delay may also increase parent stress. Prompting parents to approach a health care provider may lead to earlier intervention.

Early identification of ASD in young children is often delayed due to a mix of child and health care factors. At the child level, the varying patterns of onset and diversity in the early markers presented [5,6] can impede early identification. At the health care level, the lack of knowledge and expertise in ASD of many primary care providers and the lack of infrastructure for handling the increase in referrals further hinder early screening [7,8]. This study examined the utility of automated strategies for verifying initial parental concern in Web-based sources. In the long run, such a system can be programmed to prompt parents to seek the advice of a professional, when their concerns indicate risk.

### Parental Concerns Predict Autism Spectrum Disorder Status

Parents of children with ASD are often concerned about their child's development long before they seek professionals and receive a diagnosis [1]. Research has indicated that the type and number of early parental concerns predict ASD [2,3,6] particularly after 12 months of age [6,9]. Early concerns specific to ASD features were more prevalent among parents who already had a child with ASD [6,9] and arose earlier than in families without a previously diagnosed child with ASD [10]. The type of early concerns differentiated children later diagnosed with ASD from those with other developmental disorders [2]. The most common type of concerns are related to speech and communication [2,10]. In another study, the type and number of parental concerns correlated with Modified Checklist for Autism in Toddlers (M-CHAT) scores [11]. At the same time, for about 30% of preschool children, concerns regarding ASD did not necessarily predict an ASD diagnosis [12]; it is therefore worthwhile investigating whether specific follow-up questions would facilitate ASD prediction.

The literature reviewed points to the importance of eliciting, attending, and trusting early parental concerns in the early identification of ASD. The clinical validity of early parental concerns has been recognized by the American Academy of Pediatrics (AAP) [13] in its guidelines for the early detection of ASD in primary care. The guidelines list parental concerns related to ASD as an indicator of higher risk, and state that they should trigger ASD-specific screening at any age.

### The Internet as an Opportunity for Prescreening

Although many parents of pediatric patients use the internet to research medical information and relieve anxiety, they are likely to find incorrect/inappropriate advice [14], and only 21% share this information with their health care provider [15]. As opposed to searching, parents are turning to Web communities more and more to ask about their child’s development or health [15-17]. Parents’ high involvement and frequent access to informational exchanges on the internet offer a unique public health opportunity for promoting early screening. Furthermore, spontaneous open-ended parental concerns regarding ASD enable researchers to document an unbiased view of markers outside the scope of the standardized screening tools [1]. Advances in natural language processing and machine learning (ML) technologies offer tools for predicting ASD risk by classifying text from the growing collection of parental concerns that have been documented in Web-based sources. The long-term goal of our work is the design of an automated support system evaluating the degree of risk based on parents' concerns related to ASD markers. Such a system could prompt parents to act early or alternatively relieve their anxiety.

Evidence from other health conditions supports the effect of automated prompting on patients' health related actions such as seeking formal screening or a provider. A review of studies examining the effect of text messaging interventions on screening for cancer showed 4% to 63% higher screening rates for those who received text messages versus controls [18]. In the mental health domain, 38% of participants using a depression-screening app reported that they consulted with their health care professional about the app screening result [19]. A scoping review of studies of the impact of health information technologies for pediatric care showed, in diabetes type I studies and asthma studies, benefits for behavior change and patients' and caregivers' use of resources [20]. These diverse studies all point to the clinical utility of using automated systems to prompt health-related actions, in our case seeking formal ASD screening.

### Machine Learning Prediction From Web-Based Querying

This study builds upon research of Ben-Sasson and Yom-Tov [21], which analyzed existing queries on Yahoo Answers where parents expressed concerns regarding ASD markers in their child. The study showed that (1) parents associate a rich array of behaviors with ASD; (2) to accurately predict ASD risk status, signs mentioned in the text must be classified into symptom categories; and (3) the accuracy of automated prediction differs based on the combination of categories reported. Using ML tools, which automatically learn to distinguish between labeled examples, enabled the researchers to classify children’s risk based on expert categorization of texts into specific concern categories, though with moderate success. Area under the receiver operating curve (AUC) using the text combined with the classification of signs was 0.84 compared with 0.54 for text alone. Understanding that parental concerns alone are not sufficient for automated prediction using ML, we decided to test a prediction model that combines text analysis with structured ASD screening questions.

There is evidence that ML can use free-form text to elicit information about psychopathology markers; for example, ML has been used in the past to identify signs of postpartum depression in new mothers’ social media posts. They found an average AUC of 0.82 for predicting behavioral change of mothers between prenatal and postpartum periods [22]. There is a need for an ML-based tool that, when receiving a combination of symptoms, can assess the minimum amount of specific information needed to provide an accurate risk prediction.

**Figure 1:**
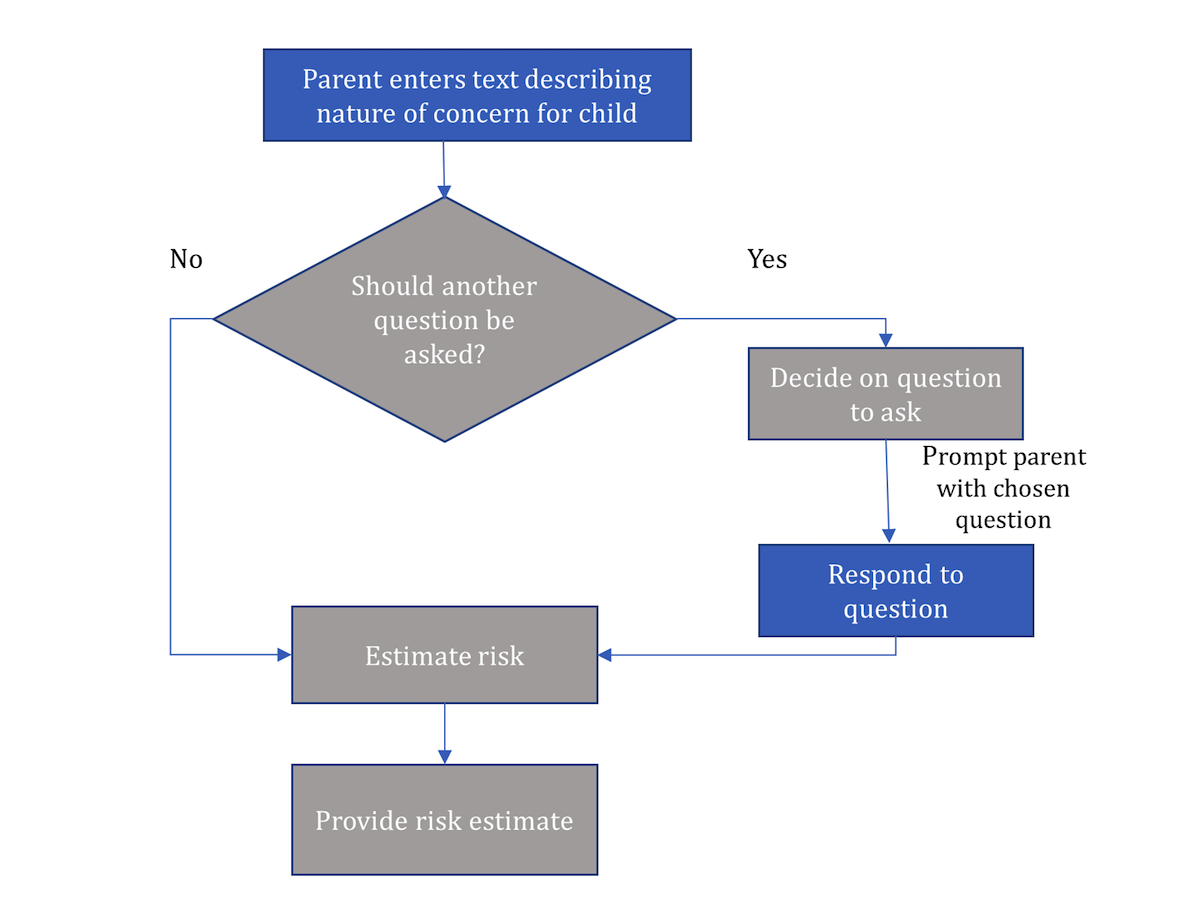
Flowchart for the proposed method of screening of parental concerns.

One of the challenges ML tools face when predicting risk from descriptions of parental concerns is their variability and individual nature, which is difficult to capture from the text of these descriptions alone. In a recent study [2], over 80 combinations of different types of signs were mentioned in parental concerns. At the same time, screening all children for a broad range of signs would entail a lengthy and costly screening process, posing a particular problem for pediatricians [7]. There is therefore a need for a mixed screening approach of user- and expert-driven report.

ML tools offer an efficient way to draw generalizations from available data. We hypothesize that these tools will be able to draw generalizations from the individual nature of parental concerns to predict ASD risk [23], so long as parents can be asked for additional information to complement their specific narrative. In this study, we wished to test whether, based on the text submitted by the parent, together with a minimal set of well-established ASD signs (M-CHAT-R questions) we could predict ASD risk. ASD risk was defined in this study by a gold standard global developmental screening tool and ASD-specific screening tools.

This study aims to (1) evaluate the ASQ and M-CHAT-R risk status of children with parental concerns in the social-communication domain, and whether this status correlates with a family history of ASD and expert assessment of ASD risk; (2) examine whether an automatically selected single M-CHAT-R question improves the accuracy of automated prediction of ASD risk based on parental concerns in Web-based sources; and (3) identify which M-CHAT-R questions contribute the most to the automated prediction of ASD risk. See Figure 1 for a description of the screening stages put forward in this study.

## Methods

### Participants

Inclusion criteria were as follows: (1) child’s age between 16 and 30 months; and (2) parent had a concern regarding their social-communication development. The final sample (n=132) included 25 parents recruited via social media and 107 recruited via Instant.ly, a service for Web-based surveys and recruitment of relevant survey participants. A total of 6 participants entered gibberish or nonsense; they were excluded from the analyses. Of all children, 7 were already diagnosed with ASD, and 4 parents did not write about their concerns but rather a different topic; they too were excluded from the analyses. The remaining 115 participants’ children had a mean age of 25.06 months (SD 4.46, range 16-30), of whom 58.3% (67/115) were boys. Of the participants, 57.4% (66/115) reported having a family member with ASD; these participants will be referred to as the ASD family group. The ASD family members included 37 siblings, 21 cousins, 7 aunts or uncles, and 1 grandson.

### Measures

We used M-CHAT-R/F [24]. The M-CHAT-R is a short gold-standard ASD-specific screening tool, in which parents of children aged 16 to 30 months rate their children’s early ASD signs. The M-CHAT-R includes 20 binary (Yes/No) questions regarding signs from the social-communication and repetitive behavior domains. Scoring of the questionnaire is based on the number of answers reporting an at-risk symptom (potential range 0-20 months); the child may be classified as low risk (0-2), medium risk (3-7), or high risk (8-20). In this study, when a parent’s answer to an item indicated risk for ASD, the structured follow-up question for that item was immediately administered. This administration method is different from the method used by others [25] who administered the initial 20 items first, later followed by the relevant follow-up items. For this study, the follow-up was programmed for administration interspersed with initial items, without first tallying a total M-CHAT-R risk status. Children whose final M-CHAT-R/F score is 2 or higher are at risk for ASD and warrant referral for diagnostic evaluation and early intervention; children whose final score is 0 to 1 are at low risk and are not in need of referral unless parents or professionals had ASD concerns.

The M-CHAT-R/F was previously validated in a sample of 16,071 low-risk toddlers [25] and showed adequate internal consistency in the 2-stage screening process (Cronbach alpha=.79). Using the threshold of 3 initial risk items to trigger follow-up, and 2 or more risk items after follow-up, they found a sensitivity of 0.85 and specificity of 0.99. Although the positive predictive value for ASD was modest (0.48), 83% (184/221) of the children who screened positive were diagnosed with a developmental disorder, and nearly 95% (209/221) demonstrated developmental delays or concerns.

Additionally, we used the Ages and Stages Questionnaire-3 (ASQ) [26]. The ASQ is a short gold-standard broad developmental screening tool, in which parents rate their child’s current skills and development. Parents answer 30 questions, 6 for each of 5 developmental domains: gross motor, fine motor, personal-social, problem solving, and communication. There are 21 versions of the ASQ for different age groups, from birth to 6 years. Items are rated by parents as “not yet” (0), “sometimes” (5), or “yes” (10). Each developmental domain yields a summary score and a risk score relative to age cutoffs. If the score is below 2 SDs from the norm, the child is considered in a need of referral. A score between 1 to 2 SDs indicates the need for monitoring in that domain. ASQ global developmental risk is defined as a score below 2 SDs in at least one domain.

### Procedures

Following ethics committee approval, participants were recruited through social media and Instant.ly. Instant.ly is a Web-based platform for conducting structured surveys, where participants are recruited based on researchers’ criteria by the company using financial incentives. All participants completed the survey on the Instant.ly survey platform after confirming that their child is between 16 to 30 months of age and that they have a concern related to their child’s social-communication development. Following this initial screening, parents were asked to describe, in their own words, their concerns regarding their child’s social-communication development. Parents answered a minimal set of background questions including age and gender of the child and whether they had a family member diagnosed with ASD. Then they answered the M-CHAT-R/F questions. Finally, parents completed the ASQ version that was appropriate for their child’s age. Parents were compensated US $35 for their participation.

To further validate the child’s risk status, we sought clinical judgment regarding the ASD risk of the child based on the parent’s narrative. A total of 3 clinicians with clinical expertise in early ASD screening rated the degree of risk of developing ASD based on reading the parents’ reported concerns, from 1 (no risk) to 4 (high risk). Raters were blind to the M-CHAT-R/F and ASQ scores. Intraclass correlation coefficient for rating ASD risk was 0.76 (95% CI 0.67-0.83).

### Data Analysis

Spearman correlations were used for correlations between standardized screening tests and with clinician ASD risk rating. Chi-square tests were conducted to compare risk status on the 3 standardized measures between the groups with a family history of ASD compared with the group without a family history of ASD.

We used the initial M-CHAT-R risk status as well as the final M-CHAT-R/F risk status to ensure that we were covering 2 scenarios. One is a parent who wants to have his/her child screened but will not see another provider for that procedure, in which case the M-CHAT-R/F is the important score. The other is a parent who wants to know whether his/her child is at risk so that they can consult with a clinician for further screening, in which case the M-CHAT-R is the relevant score.

### Machine Learning Automatic Assessment of Autism Spectrum Disorder Risk From Text

Our primary task was to predict the risk for ASD, either solely using the text provided by the parent or by augmenting the text with the parent’s response to a single question. Specifically, the algorithm for predicting risk proceeded as follows: first, the algorithm used the text provided by parents to decide which question to ask, if any. Once a response was given to the chosen question, the algorithm predicted risk.

The question to add was selected from the 20 M-CHAT-R questions. The algorithm selected which question to add (if any) based on an estimate of whether the prediction from text combined with a question is better than one based on text alone. Specifically, if we knew the risk score to a given question and were asked to choose between using the output of a text-based risk predictor and that of a text-based risk predictor augmented with an answer to the *j*-th question, we would choose the most extreme of the 2 predictor outputs. That is, if the true risk is high, we would prefer the predictor output which is higher of the two, and vice versa. In practice, we do not know the actual risk score or the answer to the *j*-th question for a test question. Therefore, we trained a second predictor to estimate whether the output of a risk predictor with an added question would give a more extreme answer (in the right direction) than that of a risk predictor without an added question. The second predictor was trained when the actual risk scores are known (in the training set), given the following independent variables: (1) output of the risk predictor using only text, (2) output of the risk predictor using text and a negative answer to the *j*-th question, (3) output of the risk predictor using text and a positive answer to the *j*-th question, and (4) variance of the 3 abovementioned predictors at the node where the test example is labeled. This procedure is repeated for all 20 augmenting questions. In the third stage, if the outputs of all selection decisions are below a particular threshold (set empirically to 0.5), we chose not to add a question to the text. If any are above that threshold, we add the question with the highest decision score.

We examined 6 possible risk outcomes: (1) ASQ, (2) M-CHAT-R, (3) M-CHAT-R/F, (4) ASQ Personal-Social, (5) ASQ Communication, and (6) Meeting ASQ Personal-Social or ASQ Communication risk. The narratives of parental concerns contained between 2 and 253 words (mean word count 72, SD 37). We represented the text of questions using single words, word pairs, and triplets, and lexical affinities [27], excluding stopwords [28], and keeping terms that appeared in 10 or more questions. The reported performance of the predictor was estimated using leave-one-out [23]. We trained the algorithm on all but one example and predicted the risk for that example, repeating the process for all examples. The algorithm used for predicting risk (using text or using text with an additional question) was a regression tree [23].

The performance of the predictors is reported using the AUC. AUC is a commonly used measure of classifier accuracy, calculated from a graph of the true-positive rate versus the false-positive rate, for different thresholds of the classifier. A perfect classifier would have an AUC of 1, whereas a random decision would achieve an AUC of 0.5.

## Results

### Risk Status Across Standardized Screening Measures

Table 1 presents the number of children who were at risk according to each of the screening measures; 79.1% (91/115) show a global developmental risk on the ASQ, and 73.9% (85/115) an ASD-specific risk on the M-CHAT-R/F. All those who were at high risk on the M-CHAT-R showed a confirmed risk on the M-CHAT-R/F. Due to the interspersed administration of the Follow-Up, fourteen children with low risk on the M-CHAT-R completed Follow-Up items. Of all the children, 14.3% (2/13) of the children at low risk and 58% (25/43) of those at medium risk on M-CHAT-R ended up categorized as at risk on the two-stage M-CHAT-R/F.

Spearman correlation coefficients between M-CHAT-R and M-CHAT-R/F total score and ASQ domain scores were significant and high (*r*_s_=−0.58 to −0.65, *P*_s_<.001). Of those at global risk according to the ASQ, 94.5% (85/91) were also at medium/high risk according to the M-CHAT-R and 82.4% (75/91) according to the M-CHAT-R/F. Only 17.6% (16/91) of those at risk based on the ASQ were not at risk on the M-CHAT-R/F, whereas 41.7% (10/42) of those who were not at risk based on the ASQ were at risk according to the M-CHAT-R/F.

Looking at ASD-related ASQ domains, 97% (65/67) with a refer score on the Personal-Social ASQ domain were at medium/high risk according to the M-CHAT-R, and 86.6% (58/67) were also at risk according to the M-CHAT-R/F. Similarly, 95.5% (64/67) with a refer score on the Communication ASQ domain were at medium/high risk on the M-CHAT-R, and 88.1% (59/67) were also at risk on to the M-CHAT-R/F.

Significant differences in odds of risk between those with versus without a family history of ASD (see Table 1) provided further validation of these screening scores. Chi-square tests indicated that those with a history of ASD in their family had significantly higher probability of risk on the M-CHAT-R and M-CHAT-R/F, as well as ASQ Personal-Social scores. Those with a history of ASD in family had nearly a 4 times higher likelihood of scoring at risk on the M-CHAT-R/F compared with those without a family history. This subgroup had a 2.63 times higher likelihood for a need for referral on the Personal-Social ASQ subscale.

**Table 1:** Modified Checklist for Autism in Toddlers-Revised (M-CHAT-R) and Ages and Stages Questionnaire (ASQ) risk rates, n (%). M-CHAT-R/F: Modified Checklist for Autism in Toddlers-Revised, with Follow-up.

Screening measures		Full sample n=115, n (%)	No ASD in family n=49, n (%)	ASD in family n=66, n (%)	Odds ratio (95% CI)	Chi-square (*df*)	*P* value
**M-CHAT-R risk**							
	M-CHAT-R medium + high risk	101 (87.8)	39 (75)	62 (93)	3.97 (1.17-13.55)	5.4 (1)	.02
	M-CHAT-R/F Risk (count fail ≥2)	85 (73.9)	29 (59)	56 (84)	3.86 (1.60-9.32)	9.6 (1)	.002
**ASQ Factors**							
	ASQ risk	91 (79.1)	36 (73)	55 (83)	1.81 (0.73-4.47)	1.7 (1)	.20
	Communication	67 (58.3)	25 (51)	42 (63)	1.68 (0.79-3.56)	1.8 (1)	.18
	Personal-Social	85 (74.0)	22 (44)	45 (68)	2.63 (1.22-5.65)	6.3 (1)	.01
	Gross motor	57 (49.6)	22 (44)	35 (53)	1.39 (.66-2.91)	0.7 (1)	.39
	Fine motor	51 (44.3)	17 (34)	34 (51)	2.00 (0.93-4.28)	3.2 (1)	.07
	Problem solving	68 (59.1)	29 (59)	39 (59)	1.00 (0.47-2.11)	0 (1)	.99

**Table 2:** Spearman correlations between screening scores and expert rating. *P* values were adjusted for multiple correlations. Ages and Stages Questionnaire (ASQ) correlations are negative as ASQ scores denote competencies whereas expert ratings reflect risk level. ASD: autism spectrum disorder; M-CHAT-R: Modified Checklist for Autism in Toddlers-Revised; M-CHAT-R/F: Modified Checklist for Autism in Toddlers-Revised, with Follow-up.

Screening measures	Expert rating of ASD risk
M-CHAT-R	0.43^a^
M-CHAT-R/F	0.36^a^
ASQ Communication	−0.21
ASQ Personal-Social	−0.26^a^
ASQ Gross Motor	−0.26^a^
ASQ Fine Motor	−0.21
ASQ Problem Solving	−0.29^a^

^a^*P*<.007.

**Table 3:** Area under the receiver operating curve (AUC) values of risk prediction. ASQ: Ages and Stages Questionnaire; M-CHAT-R: Modified Checklist for Autism in Toddlers-Revised; M-CHAT-R/F: Modified Checklist for Autism in Toddlers-Revised, with Follow-up.

Screening measures		AUC text only	AUC text + question selected
**M-CHAT-R risk**			
	M-CHAT R/F	0.39	0.85
	M-CHAT-R	0.54	0.88
**ASQ Factors**			
	ASQ Risk	0.55	0.85
	Communication	0.60	0.74
	Personal-Social	0.36	0.80
	Communication or Personal-Social	0.49	0.79

### Expert Rating of Child Risk Based on Parental Concerns

Experts rated a total of 33.9% (39/115) children, as at high (3/4) ASD risk, based on the parents’ text. M-CHAT-R, M-CHAT-R/F, and ASQ-Risk scores were all significantly associated with the experts’ rating of ASD risk (see Table 2).

### Machine Learning Automatic Assessment of Autism Spectrum Disorder Risk From Parental Concerns

Table 3 and Multimedia Appendix 1 show the proportion of children predicted as “at risk” for each of the risk measures, the AUC for risk prediction using the text of questions alone, and the AUC when a question is added. The accuracy of the predictor that selects between text-based and text-augmented predictor (the second stage predictor) had an AUC greater than 0.74 for all risk measures (10-fold cross-validation; AUCs ranged between 0.74 and 0.88 across outcome measures). Notably, M-CHAT-R initial score had the largest AUC from text and questions selected and the highest prediction from text alone (34%).

Adding a question significantly improves the accuracy of risk prediction (Friedman test, *P*=.02). The table also presents 11 single M-CHAT-R questions that when asked contributed to the prediction of risk for 5% or more of the children in at least one of the risk measures (in addition to the text). The selection of question is automatically guided by the text. Note that for the ASQ risk prediction 17% of the questions required no additional questions for estimating the children’s risk status.

## Discussion

### Principal Findings

This study examines the potential for providing automated Web-based support for parents expressing concerns regarding ASD signs in their toddler. The unique findings from this study demonstrate the potential for accurately predicting a child’s ASD risk or global development risk status from a parent’s report of concerns in Web-based forums. This prediction was made possible by supplementing open-ended concern reports with 1 question from the M-CHAT-R. Designing such a system encounters the challenges of predicting from the diverse free texts entered by parents, as well as the need to be brief. Parents are using Web-based platforms to seek advice regarding their child’s health and development [21,29]. Web-based screening for ASD is important as it may reduce the time gap between parents’ first worry and approaching a professional [3] and consequently enable the family to seek services earlier.

### Automated Support for Parents in Web-Based Forums

Findings from this study indicate that by applying ML tools, parental concerns in Web-based platofrms can be utilized for ASD prescreening. Adding a single question to the coded free response of the parents yielded AUCs ranging between 0.74 and 0.88, depending on the risk outcome measure predicted (ie, M-CHAT-R(/F), ASQ total, or specific domains). The M-CHAT-R AUCs in our study were similar to the AUC values reported previously in a study using ML tools for predicting ASD risk from parental queries combined with an ontology of the signs [21] and to the AUC in a postpartum depression screening study using ML tools [22]. The text alone predicted up to 34% of those at risk, depending on the risk outcome score predicted. These findings are consistent with research indicating that parental concerns alone cannot fully predict ASD diagnostic status [30,31]; rather, parents benefit from targeted follow-up questions to increase accuracy of prediction. Consistent with this finding, the AAP guides health care providers to follow-up on parental concerns with ASD-specific screening. The innovative ML methodology enables the selection of a single screening question, tailored based on the content of the concern, to significantly improve accuracy.

### Autism Spectrum Disorder Screening Questions That Complement Parental Concerns

Of all questions, 3 M-CHAT-R questions showed consistent contribution when combined with text to predict all 6 screening outcome measures. These items measured following a parent’s pointed finger (16%-35%), appearing deaf (9%-22%), and lack of make-believe play (6%-21%; see Table 3). Interestingly, following a pointed finger has been reported as a key question in several ASD screening studies as has lack of make-believe play [25,32] and appearing deaf [26]. However, these questions did not differentiate ASD-related risk from global developmental risk. We hypothesize that these questions inquire about behaviors that parents are less aware of and may require knowledge regarding child development to notice them. This is supported by their absence/low appearance in parental first concerns related to ASD [1,2]. However, these items were not the best added item for all parents; the diversity of ASD-related concerns reported by parents requires an individualized follow-up approach, as demonstrated by this study.

A challenge in ASD screening is differentiating global development risk from an ASD-specific risk. There is evidence that most of the false positives in ASD screening studies have a different type of developmental disorder [25,33]. The challenge of differential screening is highlighted in the significant correlations between M-CHAT-R and ASQ scores and the finding that same 3 M-CHAT-R questions contributed to ASD-specific and nonspecific outcome scores. This is in line with studies showing high correspondence between the ASQ global developmental risk and an ASD diagnosis [34]. At the same time, there was a subgroup of children who only showed risk of ASD; 41.7% (48/115) of the sample who were at risk according to the M-CHAT-R/F were not at risk according to the ASQ, whereas most of those who were at risk according to the ASQ were also at risk according to the M-CHAT-R/F. Questions that specifically predicted ASD-related risk (ie, M-CHAT-R/F, ASQ Personal-Social, ASQ Communication) and not global risk status on the ASQ were pointing to show, interest in others, showing, and responding to name. Although they added to the prediction of risk in a smaller percentage of cases, they seem to be more specific to ASD risk status. These items were among the best 7 M-CHAT-R questions for predicting ASD diagnosis [24]. As certain ASD-related questions (finger movements and getting your attention to watch him/her) improved the prediction of ASQ global risk status but did not improve the prediction of ASD-related risk status, an expert evaluation is necessary for differential diagnosis.

### Screening Risk Status for Children Whose Parents Are Concerned About Them

In absence of diagnostic outcomes, we relied upon standardized screening scores as outcome measures. Of the children in our sample, 87.8% (101/115) were at medium or high risk according to M-CHAT-R, 73.9% (85/115) according to M-CHAT-R/F, 79.1% (91/115) according to the ASQ total, and 58%-74% (20/115 to 97/115) according to the Personal-Social and/or Communication ASQ subscales. Participants in the study had concerns that likely motivated their participation. One would expect such a high positive risk rate when screening a sample of children for whom there is already concern in the social-communication domain [2,9]. Children in our study with versus without a family history of ASD had a much higher likelihood of presenting risk on ASD-specific screening and social-communication developmental screening, consistent with previous research [35,36]. It is not surprising that in a sample of parents concerned about their child’s social-communication development, there is a high rate of family history of ASD, which justifiably raises parents’ concerns given the high recurrence rates of ASD [6] and parents’ awareness of ASD symptom manifestation [9]. When interpreting the results of this study we must consider the potential impact of including concerns of parents with a family history of ASD. First, evidence indicates that siblings of children with ASD are not necessarily representative of the general ASD population [35]. Second, the language of parents with a family history of ASD may be enriched with terms that are more specific to ASD than in the general public leading to higher accuracy prediction from their text alone.

It is noteworthy that in standard use of the M-CHAT-R, some of the children in the M-CHAT-R medium-risk range eventually drop into the low-risk range after follow-up questions are administered, as opposed to the high-risk children, who are referred for evaluation without follow-up. This is consistent with prior studies [25] and suggests that in some cases, parents need additional support from the structured follow-up items to describe their child’s behavior. Hence, the M-CHAT-R/F results are more likely to predict typical versus delayed development; however, it is common to report both initial M-CHAT-R and M-CHAT-R/F outcomes.

### Limitations

Future studies should randomize the order of the M-CHAT-R and ASQ completion as the fixed order may have led to elevated ASQ scores. In addition, the interspersed administration of the M-CHAT-R/F within the M-CHAT-R was not the standardized method for administering this tool. However, this approach identified 2 children who would have been missed if the M-CHAT-R/F had been administered only to those who screened positive on the initial M-CHAT-R questionnaire. The interspersed procedure may have led to elevated risk rates on the M-CHAT-R as our risk rates were higher than those reported in another study of infant siblings of children with ASD [36]. At the same time in addition to the family history of ASD, parents in our study had specific social-communication concerns suggesting that elevated risk rates were not solely associated with measurement. In the absence of diagnostic outcomes, we cannot determine who true false-negative children were. This study requires replication with diagnostic outcomes, which would increase the clinical validity of the automated prediction and follow-up procedure. Sociodemographic features of the sample were unknown, hence further research is needed to identify potential biases in such data. In addition, to generalize results to patients in general primary care settings, this method needs to be tested with a low-risk representative sample.

### Conclusions

This study aimed to test the possibility of automatically estimating a child’s risk for ASD based on his/her parent’s description of concerns. Utilizing ML methods, we showed satisfying performance of prediction models, relying on the parent’s text and a particular M-CHAT-R question that complemented that unique text. The selection of one tailored question promoted accurate screening. Such a screening method that alerts parents can reduce the gap between first concern and approaching a health care provider. This study offers an opportunity to capitalize on digital health methods for designing a real-time support system that would ask users screening questions based on their concerns.

## Appendix

Multimedia Appendix 1 Percentage of contribution of each question to different risk measures.

## Abbreviations

AAP: American Academy of Pediatrics
ASD: Autism Spectrum Disorder
ASQ: Ages and Stages Questionnaire
AUC: area under the receiver operating curve
M-CHAT: Modified Checklist for Autism in Toddlers
M-CHAT-R/F: Modified Checklist for Autism in Toddlers-Revised, with Follow-upML: machine learning

## References

[1] Guinchat V, Chamak B, Bonniau B, Bodeau N, Perisse D, Cohen D, Danion A (2012). Very early signs of autism reported by parents include many concerns not specific to autism criteria. Res Autism Spectr Disord.

[2] Richards M, Mossey J, Robins D (2016). Parents' concerns as they relate to their child's development and later diagnosis of autism spectrum disorder. J Dev Behav Pediatr.

[3] Siklos S, Kerns K (2007). Assessing the diagnostic experiences of a small sample of parents of children with autism spectrum disorders. Res Dev Disabil.

[4] Klintwall L, Eldevik S, Eikeseth S (2015). Narrowing the gap: effects of intervention on developmental trajectories in autism. Autism.

[5] Ozonoff S, Williams BR, Landa R (2005). Parental report of the early development of children with regressive autism: the delays-plus-regression phenotype. Autism.

[6] Ozonoff S, Young GS, Steinfeld MB, Hill MM, Cook I, Hutman T, Macari S, Rogers SJ, Sigman M (2009). How early do parent concerns predict later autism diagnosis?. J Dev Behav Pediatr.

[7] Crais E, McComish C, Humphreys B, Watson LR, Baranek GT, Reznick JS, Christian RB, Earls M (2014). Pediatric healthcare professionals' views on autism spectrum disorder screening at 12-18 months. J Autism Dev Disord.

[8] Dobrez D, Sasso AL, Holl J, Shalowitz M, Leon S, Budetti P (2001). Estimating the cost of developmental and behavioral screening of preschool children in general pediatric practice. Pediatrics.

[9] Sacrey L, Zwaigenbaum L, Bryson S, Brian J, Smith I, Roberts W, Szatmari P, Roncadin C, Garon N, Novak C, Vaillancourt T, McCormick T, MacKinnon B, Jilderda S, Armstrong V (2015). Can parents' concerns predict autism spectrum disorder? A prospective study of high-risk siblings from 6 to 36 months of age. J Am Acad Child Adolesc Psychiatry.

[10] Herlihy L, Knoch K, Vibert B, Fein D (2015). Parents' first concerns about toddlers with autism spectrum disorder: effect of sibling status. Autism.

[11] Glascoe F, MacLean W, Stone W (1991). The importance of parents' concerns about their child's behavior. Clin Pediatr (Phila).

[12] Lo B, Klopper F, Barnes E, Williams K (2017). Agreement between concern about autism spectrum disorder at the time of referral and diagnosis, and factors associated with agreement. J Paediatr Child Health.

[13] Johnson C, Myers S, American Academy of Pediatrics Council on Children With Disabilities (2007). Identification and evaluation of children with autism spectrum disorders. Pediatrics.

[14] Scullard P, Peacock C, Davies P (2010). Googling children's health: reliability of medical advice on the internet. Arch Dis Child.

[15] Wainstein B, Sterling-Levis K, Baker S, Taitz J, Brydon M (2006). Use of the Internet by parents of paediatric patients. J Paediatr Child Health.

[16] Harvey S, Memon A, Khan R, Yasin F (2017). Parent's use of the Internet in the search for healthcare information and subsequent impact on the doctor-patient relationship. Ir J Med Sci.

[17] Khoo K, Bolt P, Babl F, Jury S, Goldman R (2008). Health information seeking by parents in the Internet age. J Paediatr Child Health.

[18] Uy C, Lopez J, Trinh-Shevrin C, Kwon S, Sherman S, Liang P (2017). Text messaging interventions on cancer screening rates: a systematic review. J Med Internet Res.

[19] BinDhim N, Alanazi E, Aljadhey H, Basyouni M, Kowalski S, Pont L, Shaman AM, Trevena L, Alhawassi TM (2016). Does a mobile phone depression-screening app motivate mobile phone users with high depressive symptoms to seek a health care professional's help?. J Med Internet Res.

[20] Gentles S, Lokker C, McKibbon K (2010). Health information technology to facilitate communication involving health care providers, caregivers, and pediatric patients: a scoping review. J Med Internet Res.

[21] Ben-Sasson A, Yom-Tov E (2016). Online concerns of parents suspecting autism spectrum disorder in their child: content analysis of signs and automated prediction of risk. J Med Internet Res.

[22] De Choudry M, Gamon M, Counts S, Horvitz E Predicting depression via social media. https://www.aaai.org/ocs/index.php/ICWSM/ICWSM13/paper/viewFile/6124/6351.

[23] Duda RO, Hart PE, Stork DG (2001). Pattern Classification (2nd ed.).

[24] Robins D, Fein D, Barton M (2009). Autism Speaks.

[25] Robins DL, Casagrande K, Barton M, Chen CA, Dumont-Mathieu T, Fein D (2013). Validation of the modified checklist for autism in toddlers, revised with follow-up (M-CHAT-R/F). Pediatrics.

[26] Squires J, Twombly E, Bricker D, Potter L (2009). Ages and stages questionnaires user's guide (3rd ed.).

[27] Carmel D, Farchi E, Petruschka Y, Soffer A Automatic query refinement using lexical affinities with maximal information gain.

[28] Wilbur WK, Sirotkin K (2016). The automatic identification of stop words. J Inf Sci.

[29] Plantin L, Daneback K (2009). Parenthood, information and support on the internet. A literature review of research on parents and professionals online. BMC Fam Pract.

[30] Glascoe FP, Macias MM, Wegner LM, Robertshaw NS (2007). Can a broadband developmental-behavioral screening test identify children likely to have autism spectrum disorder?. Clin Pediatr (Phila).

[31] Veness C, Prior M, Bavin E, Eadie P, Cini E, Reilly S (2012). Early indicators of autism spectrum disorders at 12 and 24 months of age: a prospective, longitudinal comparative study. Autism.

[32] Baron-Cohen S, Wheelwright S, Cox A, Baird G, Charman T, Swettenham J, Drew A, Doehring P (2000). Early identification of autism by the CHecklist for Autism in Toddlers (CHAT). J R Soc Med.

[33] Sturner R, Howard B, Bergmann P, Morrel T, Andon L, Marks D, Rao P, Landa R (2016). Autism screening with online decision support by primary care pediatricians aided by M-CHAT/F. Pediatrics.

[34] Hardy S, Haisley L, Manning C, Fein D (2015). Can screening with the Ages and Stages Questionnaire detect autism?. J Dev Behav Pediatr.

[35] Pandey J, Verbalis A, Robins DL, Boorstein H, Klin AM, Babitz T, Chawarska K, Volkmar F, Green J, Barton M, Fein D (2008). Screening for autism in older and younger toddlers with the Modified Checklist for Autism in Toddlers. Autism.

[36] Weitlauf AS, Vehorn AC, Stone WL, Fein D, Warren ZE (2015). Using the M-CHAT-R/F to identify developmental concerns in a high-risk 18-month-old sibling sample. J Dev Behav Pediatr.

