# Supplementary material for: Risk Assessment for Parents Who Suspect Their Child Has Autism Spectrum Disorder: Machine Learning Approach
Source: J Med Internet Res. 2018 Apr 24;20(4):e134. doi: 10.2196/jmir.9496 (PMC5941093; doi:10.2196/jmir.9496)
Supplement: Multimedia Appendix 1 [file jmir_v20i4e134_app1.pdf]

Appendix 1. Percentage of contribution of each question to different risk measures.

	None [a]	Q1 Follow point	Q2 Deaf	Q3 Make believe	Q4 Climb	Q5 Finger move	Q6 Point to request	Q7 Point to show	Q8 Interest in others	Q9 Show	Q10 Name	Q17 Get you to watch	Others [b] (each <5%)
**M-CHAT-R**													
M-CHAT R/F	0%	35%	22%	7%				9%			5%		22%
M-CHAT-R	34	25%	14%	10%									17%
**ASQ**													
ASQ Risk	17%	32%	13%	6%	8%	5%						7%	12%
Communication	0%	16%	11%	21%			6%	13%	8%				25%
Personal-Social	0%	28%	9%	19%	7%			9%	6%	5%			17%
Communication or Personal-Social	6%	31%	9%	16%					5%		6%		27%

Note. Percentages under the individual M-CHAT-R questions represent the percentage of children for which asking that specific question contributed to the prediction of risk status on that particular risk outcome measure. [a] None: the percentage of children for which asking no questions predicted their risk on that measure. [b] Others: the combined percentage that other M-CHAT-R questions contributed to risk prediction. Each of these questions contributed <5%.
